# Association Between the Cancer Diagnosis-to-Intervention Interval and Short-Term Analgesic Response Following Interventional Pain Treatment: A Retrospective Cohort Study

**DOI:** 10.3390/jcm15208019

**Published:** 2026-10-16

**Authors:** Mustafa Cem Yılmaz, Nevcihan Şahutoğlu Bal, Şükriye Dadalı, Şaziye Şahin, Erkan Yavuz Akçaboy

**Affiliations:** Department of Pain Medicine, Ankara Bilkent City Hospital, Ankara 06800, Türkiye

**Keywords:** cancer-related pain, interventional pain management, diagnosis-to-intervention interval, treatment response, opioid consumption, interventional procedures

## Abstract

**Background/Objectives:** Cancer-related pain remains difficult to control in a substantial proportion of patients despite contemporary pharmacological treatment, and interventional procedures may provide additional analgesic benefit. However, the relationship between the timing of these procedures and analgesic response remains insufficiently characterized. This study investigated whether the interval between cancer diagnosis and interventional pain treatment was associated with short-term analgesic response. **Methods:** We retrospectively analyzed 106 patients who underwent interventional procedures for cancer-related pain. Pain intensity was assessed using the Numeric Rating Scale (NRS), and opioid consumption was expressed as morphine equivalent daily dose (MEDD). Pain intensity was assessed at baseline, 1 week, and 1 month, whereas opioid consumption was assessed at baseline and 1 month. The primary outcome was a ≥50% reduction in NRS score at 1 month. Multivariable logistic regression was used to evaluate factors associated with response. Model assumptions, calibration, and internal validation were examined, and sensitivity analyses included generalized estimating equations, parsimonious and Firth-penalized models, and an analysis restricted to neurolytic procedures. **Results:** At 1 month, 52 patients (49.1%) achieved a ≥50% reduction in pain intensity. NRS scores decreased significantly over time (*p* < 0.001), while the reduction in MEDD at 1 month was statistically significant but modest (*p* = 0.005). Changes in NRS and MEDD were strongly correlated (ρ = 0.809, *p* < 0.001). A longer diagnosis-to-intervention interval was associated with lower odds of treatment response (OR = 0.913 per month; 95% CI, 0.853–0.977; *p* = 0.008). Metastatic disease was also associated with lower odds of response (OR = 0.067; 95% CI, 0.015–0.310; *p* < 0.001). These associations were directionally consistent across sensitivity analyses, and spline analysis showed no statistical evidence of a non-linear relationship between the diagnosis-to-intervention interval and response. **Conclusions:** A longer interval between cancer diagnosis and interventional pain treatment was associated with lower odds of short-term analgesic response. However, this interval does not represent pain duration or treatment delay and may reflect differences in disease burden and other unmeasured clinical factors. These findings should therefore be interpreted as associative rather than causal and warrant prospective investigation.

## 1. Introduction

Cancer-related pain remains a major clinical problem despite substantial advances in cancer diagnosis and treatment. Approximately 40% of patients experience pain during active treatment, with prevalence increasing to 60–70% in advanced or metastatic disease [1,2,3]. Beyond its physical burden, cancer pain is strongly associated with functional impairment, psychological distress, and diminished quality of life, reinforcing the importance of effective pain management as part of comprehensive cancer care [4,5].

Beyond oncological outcomes, persistent consequences of cancer and its treatment may also affect daily functioning, autonomy, occupational participation, and socioeconomic well-being, further emphasizing the need for comprehensive supportive and symptom-directed care [6]. Cancer-related pain is rarely driven by a single mechanism. Tumor invasion, bone destruction, neural compression, and local inflammatory processes may produce nociceptive pain, while direct nerve injury or infiltration can generate neuropathic symptoms [5,7]. Anticancer treatments, including surgery, chemotherapy, and radiotherapy, may further contribute to the overall pain burden. Consequently, many patients present with mixed pain mechanisms that may evolve over the course of their disease. This complexity is reflected in the ICD-11 classification, which recognizes chronic cancer-related pain as a distinct clinical entity and emphasizes the need for treatment strategies that take the underlying pain mechanism into account [8,9].

Pharmacological therapy remains the cornerstone of treatment and is supported by international guidelines [10,11]. However, a substantial proportion of patients continue to experience inadequate analgesia or develop dose-limiting adverse effects [1,2]. Moreover, prolonged opioid exposure may be associated with tolerance and opioid-induced hyperalgesia, further complicating pain control and potentially affecting treatment responsiveness [12]. These limitations underscore the need for multimodal strategies that extend beyond pharmacological approaches alone.

Interventional pain procedures are therefore an important component of cancer pain management, particularly in patients with refractory symptoms or intolerance to systemic therapy [13]. Neurolytic procedures targeting visceral pain pathways, including celiac plexus and splanchnic nerve neurolysis, can reduce pain intensity and opioid requirements in selected patients, particularly those with upper abdominal malignancies [14,15,16]. Neuraxial techniques, including intrathecal drug delivery, may similarly provide effective analgesia while reducing systemic opioid exposure in appropriately selected patients [17].

Despite these potential benefits, interventional procedures are often considered only after prolonged pharmacological escalation or when pain has become difficult to manage [13]. This raises an important clinical question regarding whether the interval between cancer diagnosis and interventional pain treatment is associated with subsequent treatment response. Some emerging evidence suggests that introducing interventional strategies earlier in the course of cancer pain may improve pain control and reduce cumulative opioid exposure [18]. However, direct clinical evidence examining the relationship between the cancer diagnosis-to-intervention interval and subsequent analgesic response remains limited.

The primary objective of this study was to evaluate whether the interval between cancer diagnosis and interventional pain treatment was associated with short-term analgesic response at 1 month. We hypothesized that a longer diagnosis-to-intervention interval would be associated with lower odds of achieving a ≥50% reduction in pain intensity.

## 2. Materials and Methods

### 2.1. Study Design and Ethics

This retrospective observational cohort study was conducted at the Pain Medicine Department of Ankara Bilkent City Hospital, a tertiary referral center. The protocol was approved by the institutional ethics committee (TABED 1-25-1251) and adhered to the Declaration of Helsinki. Given the retrospective design and use of de-identified data, informed consent was waived. The study was designed and reported in line with STROBE recommendations.

### 2.2. Participants and Data Collection

Adult patients (≥18 years) who underwent interventional pain procedures for cancer-related pain between January 2022 and January 2025 were screened using the institutional electronic medical record system.

Patients were included if they had cancer-related pain clinically attributable to the primary malignancy or metastatic disease and underwent at least one interventional pain procedure. Attribution of pain to the underlying malignancy was based on clinical assessment by the treating pain specialist, including the anatomical distribution of pain and relevant imaging findings. Patients whose predominant pain was attributable to cancer treatment, including postoperative pain following cancer surgery, or to a clinically significant non-cancer pain condition were excluded. Those without 1-month follow-up data were excluded, which may introduce attrition bias; however, exclusion was based solely on missing outcome data.

The diagnosis-to-intervention interval was calculated as the time, in months, from the documented date of cancer diagnosis to the date of the index interventional pain procedure. This interval does not represent the duration of cancer-related pain or the time from pain onset, opioid escalation, or referral to pain medicine, because these time points were not consistently available in the retrospective records.

To preserve independence of observations, only the first (index) procedure was included in the primary analysis. A sensitivity analysis including all procedures was performed to account for repeated interventions.

Collected variables included age, sex, cancer type, metastatic status, prior oncological treatments (chemotherapy and/or radiotherapy), baseline opioid consumption, and time from cancer diagnosis to intervention. These variables were selected based on clinical relevance and their potential to influence treatment response.

Detailed measures of performance status (e.g., ECOG or Karnofsky score), disease stage, metastatic burden and anatomical distribution, longitudinal disease trajectory, actual duration of cancer-related pain, cumulative opioid exposure, and the number or sequence of prior oncological treatment lines were not consistently available in the retrospective records and could not be incorporated reliably into cohort-level analyses. Although the presumed pain mechanism was considered clinically when selecting the intervention, pain phenotype was not systematically recorded in a standardized form suitable for cohort-level analysis.

### 2.3. Procedures

All procedures were performed by an experienced interventional pain specialist under fluoroscopic or ultrasound guidance using standard sterile techniques. The choice of procedure was based on the presumed pain mechanism, clinical presentation, anatomical distribution of pain, and routine clinical assessment.

For analysis, procedures were grouped into three categories: (1) celiac plexus neurolysis; (2) other sympathetic neurolytic procedures, including splanchnic nerve, superior hypogastric plexus, and ganglion impar neurolysis; and (3) other interventional procedures, including peripheral nerve blocks, epidural drug delivery, and intrathecal drug delivery systems.

The choice and timing of intervention were determined by the treating pain specialist on an individual basis according to clinical assessment, the presumed pain mechanism and anatomical distribution of pain, together with relevant imaging findings. Neither procedure selection nor the timing of intervention was governed by a standardized study protocol.

Celiac plexus neurolysis was performed bilaterally at the L1 vertebral level using alcohol at a concentration greater than 90% as the neurolytic agent. Superior hypogastric plexus neurolysis was similarly performed using alcohol at a concentration greater than 90%. Splanchnic nerve and ganglion impar neurolysis were performed using 6% phenol. Procedural approaches and the selection of the neurolytic technique were individualized according to the location and presumed mechanism of cancer-related pain.

Because the interventions were selected according to individual clinical indications rather than a uniform study protocol, the procedures reflected routine clinical practice. Patients were monitored after each intervention, and procedure-related adverse events documented in the medical record were systematically recorded for analysis.

### 2.4. Outcomes

Baseline NRS and opioid consumption were defined as values documented within the 7 days preceding the interventional procedure. Follow-up assessments were performed at approximately 1 week and 1 month after the procedure. Pain intensity was assessed using the Numeric Rating Scale (NRS; 0–10), where 0 indicates no pain and 10 indicates the worst imaginable pain. Opioid consumption was converted to morphine equivalent daily dose (MEDD) using the Northwest Pain Guidance Opioid Conversion Calculator [19], with opioid-specific morphine-equivalent conversion factors derived from the 2022 CDC Clinical Practice Guideline [20]. Opioids used in the cohort included oral tramadol, oral oxycodone, oral morphine, and transdermal fentanyl. Transdermal fentanyl was entered according to the prescribed delivery rate (µg/h). Methadone was not used in this cohort. The morphine-equivalent conversion factors applied were 1.0 for oral morphine, 1.5 for oral oxycodone, 0.2 for oral tramadol, and 2.4 mg/day MEDD per 1 µg/h of transdermal fentanyl delivery rate (Table S8) [20]. Post-intervention opioid dosing was not governed by a standardized tapering protocol but was individualized according to pain intensity, clinical response, and the treating physician’s assessment.

The primary outcome was a ≥50% reduction in NRS from baseline to 1 month. This threshold was considered to represent a substantial clinically important improvement, consistent with IMMPACT recommendations [21].

Secondary outcomes included:

- ≥2-point reduction in NRS at 1 month [22]
- Change in MEDD (absolute and percentage)
- Procedure-related complications

Outcome assessment relied on routinely documented clinical data, which may introduce measurement variability, although standardized recording practices were used.

### 2.5. Statistical Analysis

Statistical analyses were performed using IBM SPSS Statistics version 25 (IBM Corp., Armonk, NY, USA) and Python version 3.13 (Python Software Foundation, Wilmington, DE, USA; statsmodels version 0.14 and SciPy 1.18.1). Data distribution was assessed using the Kolmogorov–Smirnov test. Continuous variables are presented as mean ± standard deviation (SD) or median with first and third quartiles (Q1–Q3), as appropriate, and categorical variables as counts and percentages. All tests were two-sided, and a *p* value < 0.05 was considered statistically significant.

Changes in Numeric Rating Scale (NRS) scores over time were analyzed using the Friedman test followed by Dunn–Bonferroni-adjusted post hoc comparisons. The change in morphine equivalent daily dose (MEDD) between baseline and 1 month was evaluated using the Wilcoxon signed-rank test, with effect size calculated as *r* = Z/√n. The association between percentage changes in NRS and MEDD was assessed using Spearman’s rank correlation coefficient. Because percentage change in MEDD is undefined when baseline MEDD is 0 mg/day, patients with a baseline MEDD of 0 mg/day were excluded from analyses involving percentage MEDD change.

To assess potential attrition bias, baseline characteristics of patients included in the primary analysis were compared with those of patients excluded because of missing 1-month follow-up data. Continuous variables were compared using Welch’s *t*-test or the Mann–Whitney U test, as appropriate, and categorical variables using Fisher’s exact test or the Fisher–Freeman–Halton exact test. Absolute standardized mean differences (SMDs) were additionally calculated to describe the magnitude of baseline imbalance independently of sample size.

Factors associated with the primary outcome, defined as a ≥50% reduction in NRS at 1 month, were initially examined using univariable logistic regression. The primary multivariable logistic regression model included age, sex, diagnosis-to-intervention interval, primary cancer type, metastatic status, baseline MEDD, and intervention category, corresponding to 10 model parameters for 52 outcome events. Results are reported as odds ratios (ORs) with 95% confidence intervals (CIs).

Model assumptions and stability were further evaluated. Linearity in the logit for continuous predictors was assessed using the Box–Tidwell procedure. The functional form of the association between the diagnosis-to-intervention interval and the primary outcome was additionally explored using a restricted cubic spline with three knots placed at the 10th, 50th, and 90th percentiles of the interval distribution. Likelihood-ratio tests were used to evaluate the non-linear spline component and the overall association. As an additional exploratory analysis, the diagnosis-to-intervention interval was categorized as ≤6, 7–12, and >12 months to examine whether the association suggested a clinically discernible threshold. Multicollinearity was assessed using variance inflation factors (VIFs).

Given the relatively low number of outcome events in relation to the number of parameters in the primary model, additional sensitivity analyses were performed to assess the robustness of the estimates. A parsimonious logistic regression model retained the diagnosis-to-intervention interval together with metastatic status, age, baseline MEDD, and procedure type classified as neurolytic versus non-neurolytic. Firth penalized logistic regression was also performed to assess the potential impact of small-sample bias and separation. No automated variable-selection procedure was used in these sensitivity analyses. Model calibration was assessed using the Hosmer–Lemeshow goodness-of-fit test. Internal validation was performed using bootstrap resampling (500 samples) to estimate optimism-corrected AUC and calibration slope. The AUC was considered a descriptive measure of model discrimination; the models were not developed or validated for use as clinical prediction tools.

To account for the small number of patients who underwent more than one procedure, a generalized estimating equations (GEE) model with a logit link was used as a sensitivity analysis including all procedures. An additional sensitivity analysis restricted the analysis to patients who underwent neurolytic procedures (celiac plexus neurolysis or other sympathetic neurolysis). Because of the smaller number of outcome events in this subgroup, the parsimonious model specification was used.

Secondary outcomes were analyzed using the corresponding non-parametric and regression approaches. Percentage change in MEDD was compared between groups using the Mann–Whitney U or Kruskal–Wallis test, as appropriate, and associations with continuous variables were assessed using Spearman’s rank correlation. Percentage change in MEDD was further examined using multivariable linear regression. Regression assumptions and model diagnostics were evaluated using residual plots, the Shapiro–Wilk test of residuals, the Breusch–Pagan test, Cook’s distance, and leverage. Robustness of the regression coefficients was assessed using heteroscedasticity-consistent (HC3) standard errors, case-resampling bootstrap 95% CIs (2000 samples), and median (quantile) regression.

Apart from the Dunn–Bonferroni adjustment for repeated NRS comparisons, no formal adjustment for multiple testing was applied. Secondary analyses and additional sensitivity analyses were interpreted as exploratory and hypothesis-generating.

## 3. Results

### 3.1. Patient Characteristics (Table 1)

A total of 125 patients undergoing interventional procedures for cancer-related pain were initially identified. 19 patients were excluded because 1-month follow-up data were unavailable, leaving 106 patients for the primary analysis. No baseline characteristic differed statistically between included and excluded patients; however, standardized mean differences exceeded 0.20 for prior chemotherapy, prior radiotherapy, baseline NRS, baseline MEDD, primary cancer type, and intervention category, indicating potentially meaningful baseline imbalances despite the absence of statistically significant differences (Table S1).

Baseline demographic and clinical characteristics of the analyzed cohort are summarized in Table 1. The mean age was 61.7 ± 11.3 years (range, 22–87), and 61.3% of patients were male. The median interval between cancer diagnosis and interventional treatment was 9 months (range, 1–40).

**Table 1:** Baseline clinical and demographic characteristics.

	*n* = 106
Age (years), mean ± SD	61.7 ± 11.3
Male sex, *n* (%)	65 (61.3)
Diagnosis to intervention interval (months), median (min–max)	9 (1–40)
Primary cancer type, *n* (%)	
Gastrointestinal	61 (57.5)
Genitourinary	17 (16.0)
Lung	14 (13.2)
Other	14 (13.2)
Metastasis present, *n* (%)	83 (78.3)
History of chemotherapy, *n* (%)	98 (92.5)
History of radiotherapy, *n* (%)	42 (39.6)
Intervention category, *n* (%)	
Celiac plexus neurolysis	49 (46.2)
Other sympathetic neurolyses	35 (33.0)
Other procedures	22 (20.8)

SD: Standard Deviation.

Gastrointestinal malignancies predominated (57.5%), followed by genitourinary (16.0%), lung (13.2%), and other malignancies (13.2%). A detailed distribution of primary malignancies is provided in Table S7. Metastatic disease was present in 78.3% of patients; 92.5% had received prior chemotherapy and 39.6% had received prior radiotherapy.

Celiac plexus neurolysis was the most frequently performed intervention (46.2%), followed by other sympathetic neurolytic procedures (33.0%) and other interventional procedures (20.8%).

Procedure-related complications were infrequent and transient (Table 2). No complication was recorded in 100 patients (94.3%). Transient hypotension occurred in two patients following sympathetic neurolysis and in one patient following celiac plexus neurolysis, in whom it was accompanied by transient dyspnea. One patient experienced dizziness with nausea and vomiting following celiac plexus neurolysis, and catheter dislodgement occurred in two patients with neuraxial catheters. No major complication was observed.

All reported complications were transient. No major complication was observed. Overall, 6 of 106 patients (5.7%) experienced at least one complication.

### 3.2. Pain Intensity and Opioid Consumption (Table 3, Figure 1 and Figure 2)

**Figure 1:**
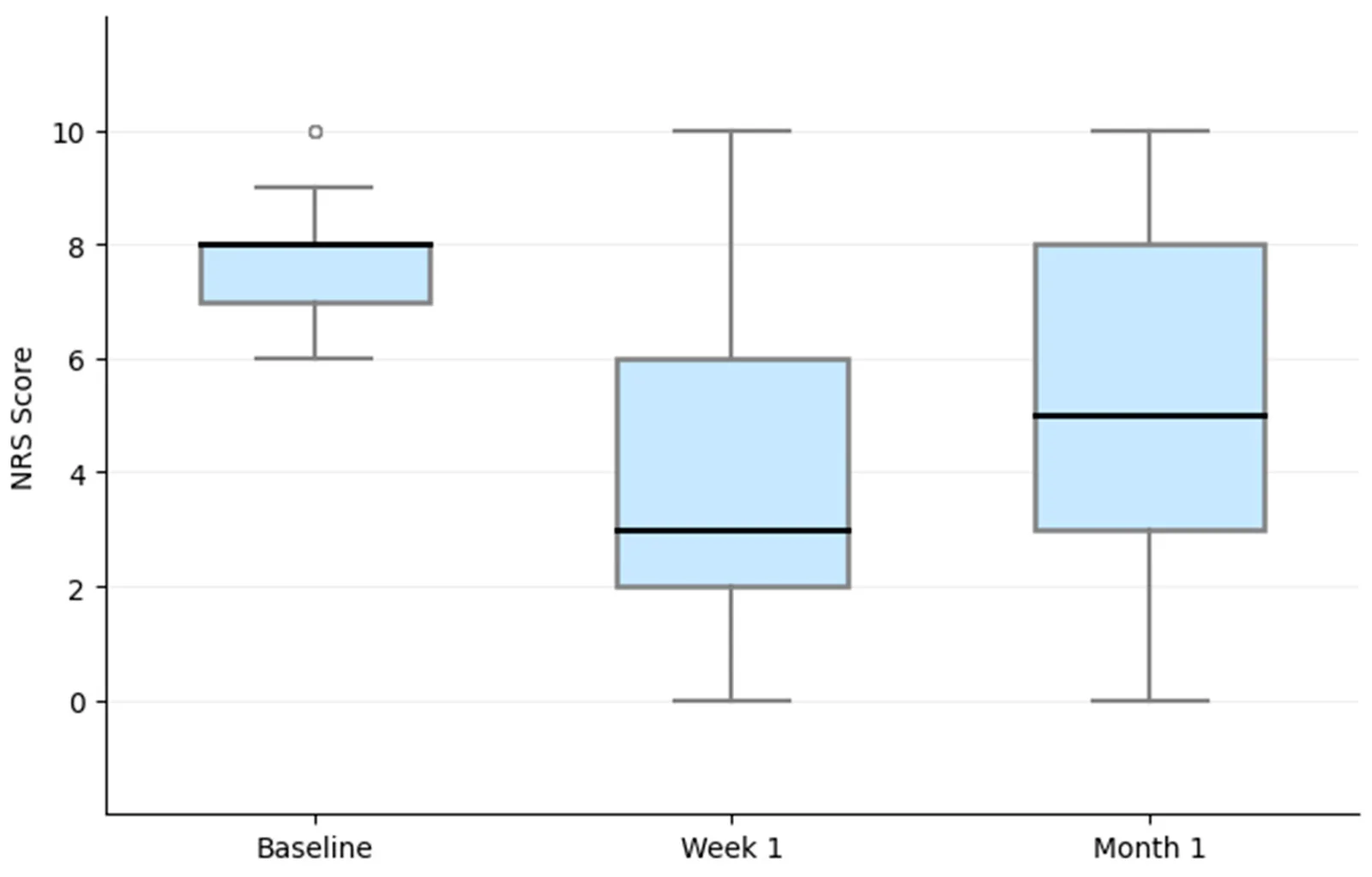
Distribution of NRS scores over time. Box plots illustrate the distribution of NRS scores at baseline, 1 week, and 1 month following interventional pain treatment. The central black line represents the median, boxes indicate the interquartile range (25th–75th percentiles), and circles denote outliers. NRS values range from 0 (no pain) to 10 (worst imaginable pain). NRS: Numeric Rating Scale.

Pain intensity decreased significantly over time (Table 3). Median NRS decreased from 8 at baseline to 3 at 1 week and 5 at 1 month (overall *p* < 0.001), with significant differences between each time point after Dunn–Bonferroni adjustment (all *p* < 0.001; Figure 1). The median change from baseline was −5 points at 1 week and −3 points at 1 month.

**Table 3:** Changes in NRS and MEDD over time.

	Baseline	1st Week	1st Month	*p*-Value
NRS score	8 (7–8) ^a,b^	3 (2–6) ^a,c^	5 (3–8) ^b,c^	<0.001 ^A^
NRS change from baseline	n/a	−5 (−6–−1)	−3 (−5–0)	n/a
% change in NRS from baseline	n/a	−62.5 (−71.4–−14.3)	−44.4 (−62.5–0)	n/a
MEDD (mg/day)	120 (60–180)	n/a	120 (40–180)	0.005 ^B^
% change in MEDD	n/a	n/a	−6.7 (−40–0)	n/a

Data were shown as median (IQR). ^A^ Friedman test, ^B^ Wilcoxon Signed-rank test. ^a^ Baseline vs. 1st week (*p* < 0.001), ^b^ Baseline vs. 1st month (*p* < 0.001), and ^c^ 1st week vs. 1st month (*p* < 0.001). n/a: Not applicable. NRS: Numeric Rating Scale; MEDD: morphine equivalent daily dose; IQR: interquartile range.

**Figure 2:**
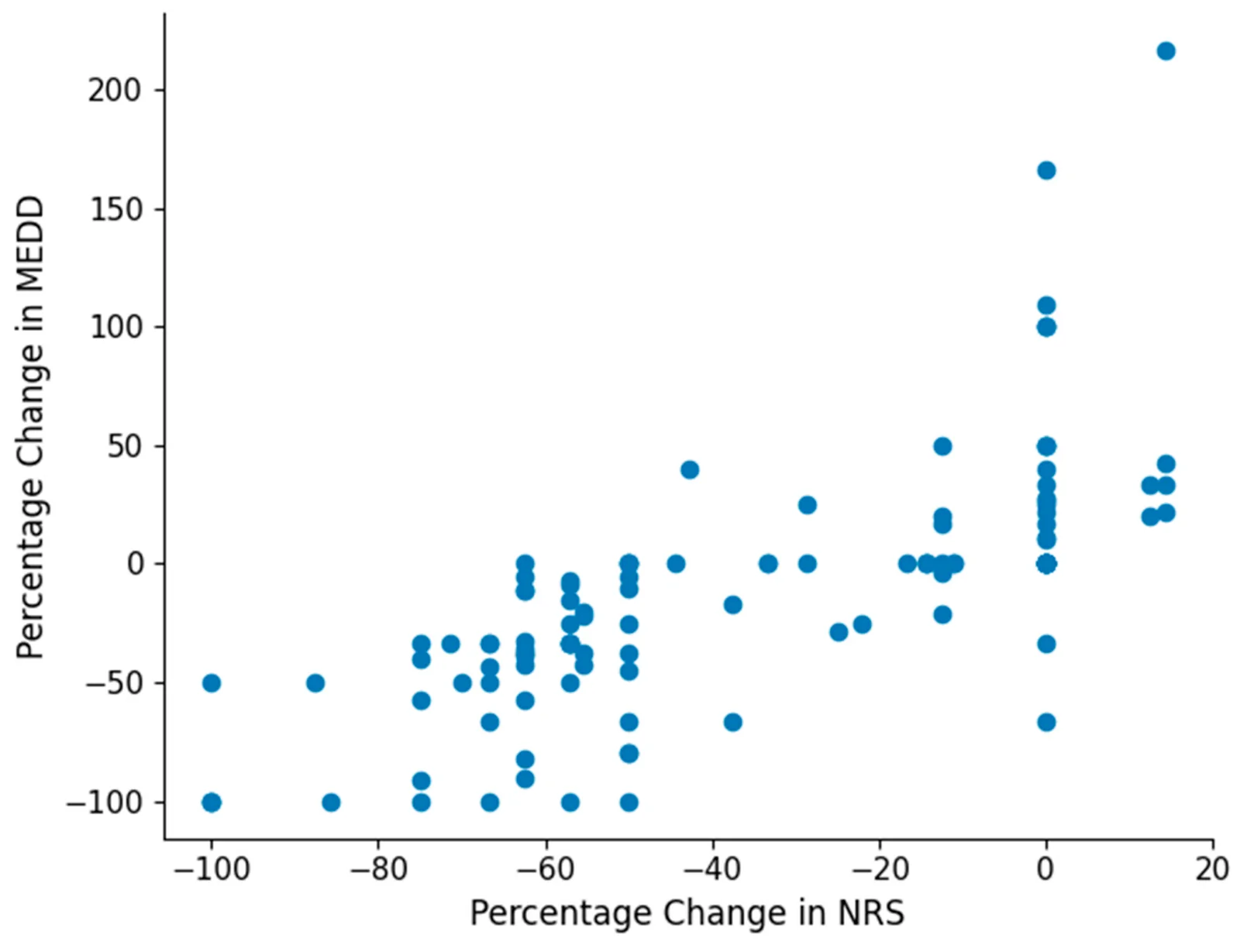
Scatter plot showing the association between percentage change in NRS and percentage change in MEDD at 1 month (*n* = 103). Correlation was assessed using Spearman’s rank correlation (ρ = 0.809, *p* < 0.001). NRS: Numeric Rating Scale; MEDD: Morphine Equivalent Daily Dose.

Median MEDD was 120 mg/day both at baseline and at 1 month; however, the within-patient change was small but statistically significant (median change, −7.5 mg/day [Q1–Q3, −40 to 0]; median percentage change, −6.7%; Wilcoxon signed-rank *p* = 0.005; effect size r = 0.27). MEDD decreased in 55 patients, remained unchanged in 25, and increased in 26. Percentage changes in NRS and MEDD were strongly correlated (Spearman ρ = 0.809, *p* < 0.001, *n* = 103; Figure 2).

### 3.3. Primary Outcome: ≥50% Pain Reduction at 1 Month (Table 4 and Table 5)

At 1 month, 52 patients (49.1%) achieved ≥50% reduction in NRS.

**Table 4:** Univariate logistic regression for ≥50% pain reduction at the 1st month.

	Non-Responder (*n* = 54)	Responder (*n* = 52)	OR (95% CI)	*p*-Value
Age (years), mean ± SD	63.0 ± 11.5	60.3 ± 10.9	0.978 (0.944–1.013)	0.211
Male sex, *n* (%)	32 (59.3)	33 (63.5)	1.194 (0.546–2.613)	0.657
Diagnosis to intervention interval (months), median (min–max)	10.5 (1–40)	7 (1–26)	0.934 (0.881–0.991)	0.024
Primary cancer type, *n* (%)				
Gastrointestinal	27 (50.0)	34 (65.4)	2.267 (0.680–7.558)	0.183
Genitourinary	12 (22.2)	5 (9.6)	0.750 (0.165–3.399)	0.709
Lung	6 (11.1)	8 (15.4)	2.400 (0.524–10.992)	0.259
Other	9 (16.7)	5 (9.6)	Reference	-
Metastasis present, *n* (%)	51 (94.4)	32 (61.5)	0.094 (0.026–0.342)	<0.001
Baseline MEDD (mg/day), median (IQR)	120 (56–180)	121.5 (60–180)	1.000 (0.996–1.004)	0.921
History of chemotherapy, *n* (%)	50 (92.6)	48 (92.3)	0.960 (0.227–4.057)	0.956
History of radiotherapy, *n* (%)	24 (44.4)	18 (34.6)	0.662 (0.302–1.449)	0.302
Intervention category, *n* (%)				
Celiac plexus neurolysis	23 (42.6)	26 (50.0)	1.633 (0.590–4.521)	0.345
Other sympathetic neurolyses	18 (33.3)	17 (32.7)	1.364 (0.464–4.008)	0.572
Other procedures	13 (24.1)	9 (17.3)	Reference	-

OR: Odds Ratio; CI: Confidence Interval.

**Table 5:** Multivariable logistic regression for ≥50% pain reduction at the 1st month.

	Odds Ratio	95% Confidence Interval		*p*-Value
		Lower Limit	Upper Limit	
Age	0.974	0.935	1.015	0.204
Male sex	0.925	0.360	2.382	0.872
Diagnosis to intervention interval	0.913	0.853	0.977	0.008
Primary cancer type				
Gastrointestinal	2.868	0.575	14.317	0.199
Genitourinary	0.835	0.136	5.142	0.846
Lung	2.936	0.510	16.921	0.228
Other	Reference	-	-	-
Metastasis present	0.067	0.015	0.310	<0.001
Baseline MEDD	1.002	0.997	1.006	0.546
Intervention category				
Celiac plexus neurolysis	0.749	0.153	3.682	0.722
Other sympathetic neurolyses	1.099	0.258	4.678	0.898
Other procedures	Reference	-	-	-

#### 3.3.1. Univariate Analysis (Table 4)

In univariable logistic regression (Table 4), a longer diagnosis-to-intervention interval was associated with lower odds of achieving a ≥50% reduction in NRS (OR = 0.934 per month; 95% CI, 0.881–0.991; *p* = 0.024). Metastatic disease was also associated with lower odds of response (OR = 0.094; 95% CI, 0.026–0.342; *p* < 0.001).

No significant associations were observed for age, sex, baseline MEDD, prior oncological treatments, cancer type, or intervention category.

#### 3.3.2. Multivariable Analysis (Table 5)

In the primary multivariable model (Table 5), a longer diagnosis-to-intervention interval remained associated with lower odds of achieving the primary outcome (OR = 0.913 per month; 95% CI, 0.853–0.977; *p* = 0.008). Metastatic disease was also associated with lower odds of response (OR = 0.067; 95% CI, 0.015–0.310; *p* < 0.001). No other covariate was statistically significant in the adjusted model.

The primary multivariable model contained 10 parameters for 52 responders (5.2 events per parameter), with a maximum VIF of 3.26. The Box–Tidwell procedure showed no statistical evidence of departure from linearity in the logit for the diagnosis-to-intervention interval (*p* = 0.591), age (*p* = 0.635), or baseline MEDD (*p* = 0.345). When the diagnosis-to-intervention interval was modeled using a restricted cubic spline, the non-linear component was not statistically significant (*p* = 0.367), whereas the overall association was significant (*p* = 0.013). The adjusted probability of response declined gradually across the observed interval without a discernible threshold (Figure S1). Given the relatively low number of events per parameter, additional sensitivity analyses were performed. Bootstrap internal validation indicated optimism in the apparent performance of the full model (apparent AUC = 0.794; optimism-corrected AUC = 0.719; optimism-corrected calibration slope = 0.657), while the corresponding estimates for the parsimonious model were 0.774, 0.734, and 0.808, respectively. Detailed model diagnostics and calibration results are provided in Table S2. Calibration of the full model was imperfect (Hosmer–Lemeshow *p* = 0.021; Table S2).

In an exploratory categorical analysis, response rates were 61.5% for diagnosis-to-intervention intervals of ≤6 months, 44.1% for 7–12 months, and 39.4% for >12 months. Compared with ≤6 months, the adjusted OR was 0.38 (95% CI, 0.12–1.20; *p* = 0.099) for 7–12 months and 0.27 (95% CI, 0.09–0.86; *p* = 0.026) for >12 months (Table S4). These categories were examined exploratorily and were not used to define an optimal intervention window.

In the parsimonious model, the diagnosis-to-intervention interval (OR = 0.915 per month; 95% CI, 0.857–0.977; *p* = 0.008) and metastatic disease (OR = 0.068; 95% CI, 0.016–0.292; *p* < 0.001) remained associated with the primary outcome. Estimates were similar with Firth penalized logistic regression (interval OR = 0.924; 95% CI, 0.866–0.986; *p* = 0.017; Table S3).

### 3.4. Sensitivity Analysis Including All Interventions (Table 6)

A total of 118 procedures were performed in 106 patients, including repeat interventions in 12 patients. In the GEE analysis accounting for within-patient clustering (Table 6), a longer diagnosis-to-intervention interval remained associated with lower odds of response (OR = 0.916 per month; 95% CI, 0.858–0.978; *p* = 0.009), as did metastatic disease (OR = 0.089; 95% CI, 0.021–0.371; *p* < 0.001).

**Table 6:** Sensitivity analysis including all interventions (GEE Model).

	Odds Ratio	95% Confidence Interval		*p*-Value
		Lower Limit	Upper Limit	
Age	0.976	0.938	1.016	0.239
Male sex	0.998	0.401	2.484	0.996
Diagnosis to intervention interval	0.916	0.858	0.978	0.009
Primary cancer type				
Gastrointestinal	2.231	0.474	10.489	0.310
Genitourinary	0.807	0.165	3.947	0.791
Lung	2.859	0.578	14.147	0.198
Other	Reference	-	-	-
Metastasis present	0.089	0.021	0.371	<0.001
Baseline MEDD	1.001	0.996	1.006	0.632
Intervention category				
Celiac Plexus Neurolysis	1.080	0.230	5.073	0.922
Other Sympathetic Neurolyses	1.171	0.305	4.493	0.817
Other procedures	Reference	-	-	-

In an additional sensitivity analysis restricted to the 84 patients who underwent neurolytic procedures (43 responders, 51.2%), a longer diagnosis-to-intervention interval remained associated with lower odds of response (OR = 0.928 per month; 95% CI, 0.866–0.994; *p* = 0.034), as did metastatic disease (OR = 0.088; 95% CI, 0.020–0.376; *p* = 0.001; Table S5).

### 3.5. Secondary Outcome: ≥2-Point NRS Reduction (Table 7 and Table 8)

At 1 month, 61 patients (57.5%) achieved a ≥2-point reduction in NRS.

**Table 7:** Univariate logistic regression for ≥2-point NRS reduction at the 1st month.

	Non-Responder (*n* = 45)	Responder (*n* = 61)	OR (95% CI)	*p*-Value
Age (years), mean ± SD	63.0 ± 11.3	60.7 ± 11.3	0.982 (0.948–1.017)	0.305
Male sex, *n* (%)	27 (60.0)	38 (62.3)	1.101 (0.500–2.426)	0.810
Diagnosis to intervention interval (months), median (min–max)	12 (1–40)	7 (1–26)	0.904 (0.849–0.963)	0.002
Primary cancer type, *n* (%)				
Gastrointestinal	22 (48.9)	39 (63.9)	3.191 (0.950–10.719)	0.061
Genitourinary	9 (20.0)	8 (13.1)	1.600 (0.375–6.820)	0.525
Lung	5 (11.1)	9 (14.8)	3.240 (0.690–15.205)	0.136
Other	9 (20.0)	5 (8.2)	Reference	-
Metastasis present, *n* (%)	42 (93.3)	41 (67.2)	0.146 (0.040–0.531)	0.003
Baseline MEDD (mg/day), median (IQR)	120 (60–180)	123 (52.5–180)	1.000 (0.996–1.004)	0.992
History of chemotherapy, *n* (%)	43 (95.6)	55 (90.2)	0.426 (0.082–2.218)	0.311
History of radiotherapy, *n* (%)	23 (51.1)	19 (31.1)	0.433 (0.195–0.960)	0.039
Intervention category, *n* (%)				
Celiac plexus neurolysis	18 (40.0)	31 (50.8)	2.067 (0.745–5.735)	0.163
Other sympathetic neurolyses	15 (33.3)	20 (32.8)	1.600 (0.547–4.681)	0.391
Other procedures	12 (26.7)	10 (16.4)	Reference	-

OR: Odds Ratio; CI: Confidence Interval.

**Table 8:** Multivariable logistic regression for ≥2-point NRS reduction at the 1st month.

	Odds Ratio	95% Confidence Interval		*p*-Value
		Lower Limit	Upper Limit	
Age	0.975	0.937	1.015	0.226
Male sex	0.927	0.347	2.477	0.879
Diagnosis to intervention interval	0.887	0.824	0.955	<0.001
Primary cancer type				
Gastrointestinal	3.389	0.648	17.710	0.148
Genitourinary	1.988	0.346	11.416	0.441
Lung	4.705	0.756	29.276	0.097
Other	Reference	-	-	-
Metastasis present	0.098	0.020	0.487	0.004
Baseline MEDD	1.001	0.996	1.006	0.660
History of radiotherapy	0.740	0.220	2.486	0.616
Intervention category				
Celiac plexus neurolysis	0.947	0.145	6.196	0.955
Other sympathetic neurolyses	1.205	0.272	5.334	0.805
Other procedures	Reference	-	-	-

#### 3.5.1. Univariate Analysis (Table 7)

In univariable analysis (Table 7), a longer diagnosis-to-intervention interval was associated with lower odds of achieving a ≥2-point reduction in NRS (OR = 0.904 per month; 95% CI, 0.849–0.963; *p* = 0.002). Metastatic disease (OR = 0.146; 95% CI, 0.040–0.531; *p* = 0.003) and prior radiotherapy (OR = 0.433; 95% CI, 0.195–0.960; *p* = 0.039) were also associated with lower odds of response.

#### 3.5.2. Multivariable Analysis (Table 8)

In the adjusted model (Table 8), a longer diagnosis-to-intervention interval remained associated with lower odds of achieving a ≥2-point reduction in NRS (OR = 0.887 per month; 95% CI, 0.824–0.955; *p* < 0.001).

Metastatic disease was also associated with lower odds of response (OR = 0.098; 95% CI, 0.020–0.487; *p* = 0.004). The association with prior radiotherapy observed in univariable analysis was no longer statistically significant after adjustment (OR = 0.740; 95% CI, 0.220–2.486; *p* = 0.616).

### 3.6. Determinants of Opioid Dose Change (Table 9, Table 10 and Table 11)

Percentage change in MEDD differed according to metastatic status (Table 9), with a greater median reduction among patients without metastatic disease than among those with metastatic disease (*p* < 0.001).

**Table 9:** Percentage change in MEDD according to clinical and disease characteristics.

	% Change in MEDD	*p*-Value
**Sex**		0.604 ^A^
Male	−10.6 (−44.1–10.3)	
Female	−5.3 (−33.3–0.0)	
**Primary cancer type**		0.553 ^B^
Gastrointestinal	−16.7 (−39.2–5.0)	
Genitourinary	−21.4 (−61.9–11.1)	
Lung	−4.2 (−36.5–34.2)	
Other	0.0 (−25.0–0.0)	
**Metastasis**		<0.001 ^A^
Absent	−37.5 (−80.9–−13.2)	
Present	0.0 (−33.3–20.6)	
**History of chemotherapy**		0.274 ^A^
No	−25.0 (−100.0–0.0)	
Yes	−5.4 (−38.2–7.5)	
**History of radiotherapy**		0.214 ^A^
No	−20.0 (−43.3–0.0)	
Yes	0.0 (−33.3–11.7)	
**Intervention category**		0.154 ^B^
Celiac Plexus Neurolysis	−10.0 (−37.5–8.3)	
Other Sympathetic Neurolyses	−29.2 (−64.3–0.0)	
Other procedures	0.0 (−18.7–11.7)	

Data were shown as median (IQR). ^A^ Mann–Whitney U test and ^B^ Kruskal–Wallis test.

Percentage change in MEDD showed modest positive correlations with age (ρ = 0.244; *p* = 0.013) and the diagnosis-to-intervention interval (ρ = 0.246; *p* = 0.012; Table 10).

**Table 10:** Correlation analysis between percentage change in MEDD and clinical variables.

	Coefficient of Correlation	*p*-Value ^A^
Age	0.244	0.013
Diagnosis to intervention interval	0.246	0.012
Baseline NRS	0.041	0.682

^A^ Spearman’s correlation analysis.

In multivariable linear regression (Table 11), the diagnosis-to-intervention interval (B = 1.823; 95% CI, 0.370–3.276; *p* = 0.014), age (B = 0.933; 95% CI, 0.084–1.781; *p* = 0.032), and metastatic disease (B = 38.085; 95% CI, 14.131–62.039; *p* = 0.002) were associated with percentage change in MEDD.

Residuals from the linear regression model departed from normality (Shapiro–Wilk *p* < 0.001), whereas there was no statistical evidence of heteroscedasticity (Breusch–Pagan *p* = 0.570). No observation had a Cook’s distance >1, although four observations exceeded the 4/n screening threshold (Figure S2; Table S6). Estimates for the diagnosis-to-intervention interval, age, and metastatic disease were similar when HC3 robust standard errors and bootstrap confidence intervals were used. However, in median regression, only metastatic disease remained statistically significant (B = 32.8; 95% CI, 9.2–56.4; *p* = 0.007), whereas the associations with the diagnosis-to-intervention interval (B = 1.25; *p* = 0.088) and age (B = 0.48; *p* = 0.258) were attenuated and no longer statistically significant. These secondary findings should therefore be interpreted as exploratory.

**Table 11:** Multivariable linear regression analysis for percentage change in MEDD.

	Coefficient of Regression (B)	95% Confidence Interval for B		*p*-Value
		Lower Limit	Upper Limit	
Age	0.933	0.084	1.781	0.032
Male sex	−3.652	−23.340	16.036	0.714
Diagnosis to intervention interval	1.823	0.370	3.276	0.014
Metastasis present	38.085	14.131	62.039	0.002

## 4. Discussion

In this retrospective cohort, a longer interval between cancer diagnosis and interventional pain treatment was associated with lower odds of achieving a ≥50% reduction in pain intensity at 1 month. Importantly, the diagnosis-to-intervention interval examined in this study should not be interpreted as the duration of cancer-related pain or as a direct measure of delay in receiving interventional pain treatment. Pain intensity decreased substantially during follow-up, whereas the overall reduction in opioid consumption was statistically significant but modest; changes in pain intensity and opioid consumption were strongly correlated. Although pain intensity improved substantially at 1 week, the increase in median NRS from 3 at 1 week to 5 at 1 month suggests partial attenuation of the initial analgesic improvement, while pain intensity remained lower than at baseline. Accordingly, the present findings should be interpreted as evidence of short-term analgesic response and do not establish the durability of benefit beyond 1 month. The association between a longer diagnosis-to-intervention interval and lower odds of pain response was directionally consistent across sensitivity analyses, including parsimonious, penalized, GEE, and neurolytic-only models.

The observed association between the diagnosis-to-intervention interval and pain response should be interpreted within the broader context of disease trajectory and clinical complexity. A longer interval from cancer diagnosis to intervention may coincide with greater disease burden, progression of metastatic disease, cumulative oncological treatment, changes in functional status, or increasing complexity of pain mechanisms. Metastatic disease was associated with substantially lower odds of short-term pain response after multivariable adjustment. The magnitude of this association should, however, be interpreted cautiously. Metastatic status may reflect several interrelated features, including greater disease burden, multiple or evolving pain generators, functional deterioration, and cumulative oncological treatment exposure. Because the number and location of metastases, disease progression, performance status, and systematically characterized pain phenotype were not available, the present data cannot determine which of these factors contributed to the observed association. Accordingly, the finding should not be interpreted as evidence that metastatic disease itself directly reduces the efficacy of interventional pain procedures. Thus, the diagnosis-to-intervention association may partly reflect unmeasured differences in disease severity and clinical complexity rather than the effect of elapsed time itself.

Because the decision to intervene and the timing of intervention were based on clinical judgment rather than a standardized protocol, confounding by indication cannot be excluded. Patients treated at different diagnosis-to-intervention intervals may therefore have differed in unmeasured clinical characteristics that could also influence treatment response.

Cancer-related pain remains common throughout the disease trajectory and becomes particularly prevalent in advanced and metastatic disease [1,2,3]. Its management is complicated by the coexistence of nociceptive, neuropathic, and inflammatory mechanisms, together with psychological and functional factors that may further increase symptom burden [5,7]. This biological complexity is further compounded by psychological distress and functional decline, both of which amplify symptom burden and adversely affect quality of life [4]. Although pharmacological treatment, particularly opioid-based therapy, remains central to cancer pain management, adequate analgesia is not achieved in every patient, and dose escalation may be limited by adverse effects [10,11,23]. These limitations provide the rationale for incorporating interventional techniques into multimodal treatment when systemic therapy alone is insufficient.

Interventional pain techniques have therefore become an integral component of multimodal cancer pain management. Neurolytic interventions targeting visceral pathways -particularly celiac plexus and splanchnic nerve neurolysis- have demonstrated consistent efficacy in reducing pain intensity, as supported by systematic reviews and meta-analyses [14,15,16]. These approaches are also endorsed by contemporary consensus recommendations, including those of the American Society of Pain and Neuroscience [13], and may be complemented in selected patients by other advanced strategies such as intrathecal drug delivery systems [17,24]. In the present cohort, 49.1% of patients achieved at least a 50% reduction in pain intensity at 1 month, while 57.5% achieved a reduction of at least 2 NRS points. In our cohort, changes in pain intensity and opioid dose were strongly correlated. However, because post-intervention opioid dosing was individualized partly according to pain intensity and clinical response, these outcomes are not independent. The observed association therefore indicates that opioid dose changes occurred in parallel with changes in pain intensity and should not be interpreted as evidence of an independent or substantial opioid-sparing effect. Previous studies have reported reductions in opioid requirements following selected interventional cancer pain procedures; however, the magnitude and clinical relevance of opioid reduction in the present cohort were modest and should be interpreted cautiously [13,25].

The clinical heterogeneity of the interventions should also be considered when interpreting these findings. The cohort included procedures with different indications, anatomical targets, mechanisms of action, and expected duration of effect, and procedure selection was individualized according to the clinical presentation rather than assigned according to a standardized protocol. Although intervention category was included in the primary multivariable model, such adjustment cannot fully account for confounding by indication. In the sensitivity analysis restricted to neurolytic procedures, the association between a longer diagnosis-to-intervention interval and lower odds of pain response remained directionally consistent with the primary analysis. However, this finding does not eliminate procedural heterogeneity, including differences among neurolytic targets, and the sample size did not permit reliable procedure-specific multivariable analyses.

Primary malignancy may represent an additional source of clinical heterogeneity. Although the broadly grouped cancer-type variable was not statistically associated with the primary outcome, individual malignancies differed substantially in anatomical distribution, pain mechanisms, and the interventions for which patients were selected. The relatively small numbers within individual tumor types precluded reliable cancer-specific multivariable analyses; therefore, the absence of an association for the grouped cancer-type variable should not be interpreted as evidence that primary malignancy has no influence on treatment response.

A principal finding of this study was the association between the diagnosis-to-intervention interval and short-term pain response. In many real-world settings, interventional procedures are introduced relatively late, often following prolonged pharmacological escalation [10,14,26,27]. More recent approaches to cancer pain management have questioned whether interventions should be reserved exclusively for these later stages, and emerging literature has suggested potential advantages of integrating selected interventional techniques earlier in the treatment course [18,28]. In our cohort, a longer diagnosis-to-intervention interval was associated with lower odds of achieving both the primary and secondary pain outcomes, and a similar direction of association was observed when repeated procedures were included using GEE. Although the association was summarized using a per-month odds ratio, complementary spline and exploratory categorical analyses showed no statistical evidence of a non-linear relationship and did not identify a clinically supported threshold or optimal intervention window. Accordingly, the per-month estimate should be interpreted as a summary of the observed association rather than as evidence of a uniform biological effect for each additional month or of a benefit from earlier intervention.

Several mechanisms could plausibly contribute to this association, although they cannot be established from the present data. Persistent nociceptive input can promote peripheral and central sensitization, including increased excitability within spinal nociceptive pathways and amplification of pain signaling [29,30]. Over time, these changes may evolve into maladaptive neuroplastic states, in which pain becomes less dependent on peripheral input and more resistant to targeted interventions. In parallel, prolonged opioid exposure may contribute to tolerance and opioid-induced hyperalgesia, further altering pain processing and diminishing responsiveness to both pharmacological and interventional therapies [12,31]. These mechanisms provide a biologically plausible hypothesis for the observed association between a longer diagnosis-to-intervention interval and lower probability of response; however, the present data cannot determine whether these mechanisms account for that association. Prospective studies incorporating actual pain duration, opioid exposure, and mechanistic pain phenotyping are required to examine this hypothesis directly.

Disease progression may provide another explanation. Patients with advanced cancer often develop several concurrent pain generators, including visceral involvement, bone metastases, neural infiltration, and treatment-related pain [32,33]. These mechanisms give rise to complex mixed pain phenotypes involving both nociceptive and neuropathic components [7,31,34], which are typically more difficult to treat and may respond less favorably to targeted interventions. Metastatic disease was associated with substantially lower odds of short-term pain response after adjustment for the measured covariates, although the magnitude of this association should be interpreted cautiously. Previous studies of celiac plexus neurolysis have reported heterogeneous disease-related correlates of analgesic response: distant metastatic disease has been associated with poorer response, whereas performance status, baseline opioid exposure, and specific anatomical patterns of metastatic involvement have also been associated with treatment outcome [35,36]. In our cohort, metastatic status was recorded only as a binary variable and did not capture metastatic burden or distribution, disease progression, performance status, or pain phenotype. It may therefore represent a surrogate for broader disease severity and clinical complexity rather than an isolated biological determinant of procedural efficacy.

The relationship between analgesic response and opioid consumption is also clinically relevant. Although opioids remain indispensable for many patients with moderate-to-severe cancer pain, prolonged treatment may be accompanied by sedation, gastrointestinal adverse effects, cognitive impairment, endocrine effects, and other treatment burdens [31,34]. Consequently, strategies that achieve analgesia while reducing opioid exposure are of considerable clinical importance. Changes in pain intensity and opioid dose were strongly correlated; however, because opioid dosing was adjusted according to clinical response, this relationship should be interpreted as parallel changes rather than as evidence of an independent opioid-sparing effect. This is consistent with emerging literature showing that interventional techniques not only improve pain control but also contribute to reductions in opioid burden [25]. Older age was also associated with a less pronounced reduction in MEDD in the ordinary least-squares analysis; however, this association was attenuated and no longer statistically significant in median regression and should therefore be regarded as an exploratory and tentative finding.

Treatment timing and metastatic status may therefore provide clinically relevant information when evaluating patients for interventional pain treatment. However, the model was developed in a relatively small, single-center cohort and was not intended or validated as a clinical prediction tool. External validation in larger populations would be required before these variables could be incorporated into formal risk-stratification approaches.

From a clinical perspective, the present findings identify the diagnosis-to-intervention interval as a factor associated with short-term pain response, but they do not establish that advancing the timing of an indicated interventional procedure would improve outcomes. Whether earlier interventional treatment provides greater benefit should be evaluated prospectively using actual pain duration, referral timing, disease trajectory, and predefined intervention-timing strategies [10,18].

Several limitations should be acknowledged. The retrospective design precludes causal inference and introduces potential selection and information bias. Exclusion of patients without 1-month follow-up data may have introduced attrition bias, and reliance on routinely documented clinical data may have resulted in some measurement variability. Residual confounding, particularly related to disease severity, functional status, tumor burden, and cumulative opioid exposure, cannot be fully excluded despite multivariable adjustment. Importantly, the interval examined in this study was the time from cancer diagnosis to intervention and should not be interpreted as the duration of cancer-related pain itself, which could not be reliably determined from the available retrospective data. The cohort included clinically heterogeneous interventional procedures, and both procedure selection and the timing of intervention were individualized according to clinical judgment rather than governed by a standardized protocol, introducing the possibility of confounding by indication. Although intervention category was included in the adjusted model and the main association remained directionally consistent in an analysis restricted to neurolytic procedures, the sample size did not permit reliable procedure-specific analyses, and residual procedural heterogeneity therefore remains an important limitation. Primary malignancies were also grouped into broad categories for multivariable analysis because of the limited number of patients within individual tumor types. Consequently, the study had limited ability to evaluate tumor-specific differences in treatment response, and a clinically relevant influence of primary malignancy cannot be excluded despite the absence of a statistically significant association for the grouped cancer-type variable. Follow-up was limited to 1 month; therefore, the durability of analgesic benefit and longer-term changes in opioid requirements could not be assessed. This limitation is particularly relevant given the heterogeneity of the included procedures and their potentially different durations of effect.

The primary multivariable model contained 10 parameters for 52 responders, resulting in a relatively low events-per-parameter ratio, wide confidence intervals and limited precision for several cancer-type and procedure-category estimates, and a risk of model overfitting. Internal validation by bootstrap resampling indicated optimism in the apparent model performance. Although the association between the diagnosis-to-intervention interval and the primary outcome remained directionally consistent in parsimonious and Firth-penalized models, alternative functional-form analyses, GEE, and the neurolytic-only sensitivity analysis, these findings do not eliminate the possibility of model instability or residual confounding. The models were developed to examine associations rather than for clinical prediction; no external validation was performed, and their discrimination and calibration should therefore not be interpreted as externally validated predictive performance.

The diagnosis-to-intervention interval was measured from the date of cancer diagnosis rather than from the onset of cancer-related pain, escalation of analgesic treatment, or referral to pain medicine. Reliable information on these time points was not consistently available retrospectively. Consequently, patients with similar diagnosis-to-intervention intervals may have had substantially different pain trajectories, and this interval should not be interpreted as pain duration or as a direct measure of treatment delay. Therefore, the observed association does not establish that postponing an indicated interventional pain procedure reduces treatment efficacy.

Detailed measures of performance status (e.g., ECOG or Karnofsky score), disease stage, metastatic burden and location, longitudinal disease trajectory, actual duration of cancer-related pain, systematically characterized pain phenotype, and cumulative opioid exposure were not available. Although pain mechanism was considered clinically when selecting the interventional procedure, it was not systematically recorded in a form suitable for cohort-level phenotypic analysis. Metastatic status and several measured clinical characteristics were included in the adjusted analyses; however, these variables cannot fully characterize disease severity, functional status, pain complexity, or prior treatment burden. Residual confounding by these unmeasured factors therefore remains possible.

In addition, no formal adjustment for multiplicity was applied to secondary and exploratory analyses. Accordingly, these findings should be regarded as hypothesis-generating. In particular, the associations of the diagnosis-to-intervention interval and age with percentage change in MEDD were attenuated and no longer statistically significant in median regression, and should therefore be interpreted cautiously.

Finally, 19 of 125 potentially eligible patients (15.2%) were excluded because 1-month follow-up data were unavailable. Although no baseline characteristic differed statistically between included and excluded patients, standardized mean differences indicated potentially meaningful imbalances in several characteristics. Moreover, the reasons for missing follow-up could not be fully characterized. Consequently, the possibility, direction, and magnitude of attrition bias cannot be determined and should be considered when interpreting the findings.

Despite these limitations, the study has several strengths. Clinically meaningful responder definitions were used rather than relying only on changes in mean pain scores, opioid consumption was evaluated alongside analgesic response, and the principal associations were examined using both index-procedure and repeated-procedure analyses. The consistency of the association between the diagnosis-to-intervention interval and response across these analyses makes this observation worthy of further prospective investigation. Future studies should specifically examine pain duration, cumulative opioid exposure, performance status, disease burden, and pain phenotype to determine whether an optimal window for interventional treatment can be identified.

## 5. Conclusions

In this retrospective cohort, interventional pain treatment was followed by substantial short-term reductions in pain intensity, whereas the overall reduction in opioid consumption was statistically significant but modest. Metastatic disease was also associated with lower odds of short-term pain response, although this finding may partly reflect unmeasured differences in disease burden and clinical complexity. A longer interval between cancer diagnosis and interventional pain treatment was associated with lower odds of short-term pain response after adjustment for measured covariates. However, this interval should not be interpreted as pain duration or treatment delay, and the observational design does not establish that earlier intervention improves treatment outcomes. Prospective studies incorporating actual pain duration, disease burden, performance status, pain phenotype, and predefined timing of intervention are needed to clarify the clinical relevance of this association.

## Figures and Tables

**Table 2:** Procedure-related complications according to intervention category.

	Celiac Plexus Neurolysis (*n* = 49)	Other Sympathetic Neurolyses (*n* = 35)	Other Procedures (*n* = 22)	Total (*n* = 106), *n* (%)
No complication	47	33	20	100 (94.3)
Transient hypotension	0	2	0	2 (1.9)
Transient hypotension with dyspnea	1	0	0	1 (0.9)
Dizziness, nausea and vomiting	1	0	0	1 (0.9)
Catheter dislodgement	0	0	2	2 (1.9)

## Data Availability

The data presented in this study are available from the corresponding author upon reasonable request. The data are not publicly available because they contain clinical information subject to patient privacy and institutional data-protection requirements.

## Supplementary Materials

The following supporting information can be downloaded at: https://www.mdpi.com/article/10.3390/jcm15208019/s1, File S1 (STROBE Checklist); Tables S1–S8 and Figures S1 and S2.
