# Supplementary material for: Association Between the Cancer Diagnosis-to-Intervention Interval and Short-Term Analgesic Response Following Interventional Pain Treatment: A Retrospective Cohort Study
Source: J Clin Med. 2026 Oct 16;15(20):8019. doi: 10.3390/jcm15208019 (PMC13706139; doi:10.3390/jcm15208019)
Supplement: Supplementary file 1 [file jcm-15-08019-s001.zip › jcm-4555018-supplementary figures and tables.pdf]

Revision tables and figure legends

Table S1. Baseline characteristics of patients included in the primary analysis and of patients excluded because of missing 1-month follow-up.

	Included (n=106)	Excluded (n=19)	SMD	p-value
Age (years)	61.7 ± 11.3	61.3 ± 12.4	0.03	0.909
Male sex	65 (61.3)	11 (57.9)	0.07	0.803
Primary cancer type			0.43	0.544
Gastrointestinal	61 (57.5)	14 (73.7)		
Genitourinary	17 (16.0)	3 (15.8)		
Lung	14 (13.2)	1 (5.3)		
Other	14 (13.2)	1 (5.3)		
Metastatic disease	83 (78.3)	16 (84.2)	0.15	0.762
Prior chemotherapy	98 (92.5)	19 (100.0)	0.40	0.606
Prior radiotherapy	42 (39.6)	4 (21.1)	0.41	0.195
Baseline NRS	8 (7–8)	8 (8–9)	0.35	0.167
Baseline MEDD (mg/day)	120 (60–180)	100 (60–135)	0.31	0.336
Diagnosis-to-intervention interval (months)	9 (5–15)	10 (7–14)	0.13	0.478
Intervention category			0.28	0.576
Celiac plexus neurolysis	49 (46.2)	10 (52.6)		
Other sympathetic neurolyses	35 (33.0)	7 (36.8)		
Other procedures	22 (20.8)	2 (10.5)		

Data are mean ± SD, median (Q1–Q3) or n (%). Age was compared with Welch's t-test; baseline NRS, baseline MEDD and the diagnosis-to-intervention interval with the Mann-Whitney U test; binary variables with Fisher's exact test; primary cancer type and intervention category with the Fisher-Freeman-Halton exact test (Monte Carlo, 20,000 samples). SMD: absolute standardized mean difference (for multi-level variables the Mahalanobis-type multivariate SMD of Yang and Dalton); values below 0.20 are conventionally regarded as negligible imbalance. MEDD: morphine equivalent daily dose; NRS: Numeric Rating Scale.

Table S2. Specification, diagnostics and internal validation of the multivariable logistic regression models for ≥50% NRS reduction at 1 month.

	Full model (Table 4)	Parsimonious model (Table S3)
Model parameters (excluding intercept)	10	5
Events (responders) / events per parameter	52 / 5.2	52 / 10.4
Maximum variance inflation factor	3.26	1.06
Linearity in the logit, Box-Tidwell test (p-value)	interval 0.591; age 0.635; baseline MEDD 0.345	–
Restricted cubic spline for the interval (3 knots): p for non-linearity / p for overall association	0.367 / 0.013	–
Nagelkerke R^2	0.348	–
Hosmer-Lemeshow test, χ^2 (df = 8), p-value	18.05, 0.021	2.03, 0.980
Apparent AUC (95% CI)	0.794 (0.700–0.873)	0.774
Bootstrap optimism in AUC / optimism-corrected AUC	0.075 / 0.719	0.040 / 0.734
Optimism-corrected calibration slope	0.657	0.808

Internal validation by bootstrap resampling (500 samples; the model was refitted in each bootstrap sample and evaluated in the original data; optimism = bootstrap performance minus original-data performance, averaged over samples). Bootstrap samples with non-convergence or complete separation were discarded (37 and 23 samples, respectively). The apparent calibration slope is 1 by construction and is therefore not shown. Spline knots at the 10th, 50th and 90th percentiles of the interval (2, 9 and 20.5 months). AUC: area under the receiver operating characteristic curve; CI: confidence interval; MEDD: morphine equivalent daily dose; NRS: Numeric Rating Scale.

Table S3. Multivariable logistic regression for ≥50% NRS reduction at 1 month: full model, parsimonious model and Firth penalized model.

	Full model OR (95% CI)	p	Parsimonious model OR (95% CI)	p	Firth model OR (95% CI)	p
Diagnosis-to-intervention interval (per month)	0.913 (0.853–0.977)	0.008	0.915 (0.857–0.977)	0.008	0.924 (0.866–0.986)	0.017
Metastatic disease	0.067 (0.015–0.310)	<0.001	0.068 (0.016–0.292)	<0.001	0.097 (0.024–0.387)	<0.001
Age (per year)	0.974 (0.935–1.015)	0.204	0.975 (0.937–1.014)	0.197	0.977 (0.938–1.016)	0.244
Baseline MEDD (per mg/day)	1.002 (0.997–1.006)	0.546	1.001 (0.997–1.006)	0.511	1.001 (0.997–1.006)	0.589
Male sex	0.925 (0.360–2.382)	0.872	–	–	0.937 (0.373–2.354)	0.890
Neurolytic procedure (vs other procedures)	–	–	1.040 (0.366–2.959)	0.941	–	–
Celiac plexus neurolysis (vs other procedures)	0.749 (0.153–3.682)	0.722	–	–	0.771 (0.164–3.628)	0.742
Other sympathetic neurolyses (vs other procedures)	1.099 (0.258–4.678)	0.898	–	–	1.078 (0.266–4.372)	0.916
Gastrointestinal cancer (vs other)	2.868 (0.575–14.317)	0.199	–	–	2.440 (0.517–11.524)	0.260
Genitourinary cancer (vs other)	0.835 (0.136–5.142)	0.846	–	–	0.845 (0.148–4.829)	0.850
Lung cancer (vs other)	2.936 (0.510–16.921)	0.228	–	–	2.477 (0.453–13.533)	0.295

The full model is the model reported in Table 4. The parsimonious model includes the exposure and core covariates selected on clinical grounds (metastatic status, age, baseline MEDD, and neurolytic versus non-neurolytic procedure) and was not derived by univariable screening or automated stepwise selection; likelihood-ratio test of the full against the parsimonious model p = 0.530. The Firth model applies Jeffreys-prior penalization to the full specification (Wald confidence intervals). CI: confidence interval; MEDD: morphine equivalent daily dose; OR: odds ratio.

Table S4. Treatment response according to categories of the diagnosis-to-intervention interval.

Interval category	n	Responders, n (%)	Adjusted OR (95% CI)	p-value
≤6 months	39	24 (61.5)	Reference	–
7–12 months	34	15 (44.1)	0.380 (0.121–1.198)	0.099
>12 months	33	13 (39.4)	0.272 (0.086–0.856)	**0.026**

Adjusted for the covariates of the full model (age, sex, primary cancer type, metastatic status, baseline MEDD, intervention category). Categories were selected on clinical grounds for exploratory assessment of the shape of the association; they do not represent a derived or validated cut-off. CI: confidence interval; MEDD: morphine equivalent daily dose; OR: odds ratio.

Table S5. Sensitivity analysis restricted to neurolytic procedures (n = 84; 43 responders, 51.2%): multivariable logistic regression for ≥50% NRS reduction at 1 month.

	**OR (95% CI)**	**p-value**
Diagnosis-to-intervention interval (per month)	0.928 (0.866–0.994)	**0.034**
Metastatic disease	0.088 (0.020–0.376)	**0.001**
Age (per year)	0.958 (0.915–1.002)	0.063
Baseline MEDD (per mg/day)	1.001 (0.995–1.006)	0.786
Celiac plexus neurolysis (vs other sympathetic neurolyses)	1.312 (0.433–3.974)	0.631

Patients who underwent celiac plexus neurolysis or other sympathetic neurolytic procedures (intervention categories 1 and 2). A parsimonious specification was used because of the smaller number of events (5 parameters; 8.6 events per parameter). Unadjusted OR for the interval in this subgroup: 0.939 (0.881–1.002), p = 0.056. CI: confidence interval; MEDD: morphine equivalent daily dose; OR: odds ratio.

Table S6. Multivariable linear regression for percentage change in MEDD (Table 10): regression diagnostics and robustness analyses (n = 103).

	OLS B (95% CI)	p	HC3 robust 95% CI	p	Bootstrap 95% CI	Median regression B (95% CI)	p
Age (per year)	0.93 (0.08–1.78)	0.032	0.16–1.71	0.018	0.26–1.80	0.48 (-0.36–1.32)	0.258
Male sex	-3.65 (-23.34–16.04)	0.714	-23.17–15.87	0.714	-23.55–14.53	9.00 (-10.40–28.41)	0.359
Diagnosis-to-intervention interval (per month)	1.82 (0.37–3.28)	0.014	0.30–3.35	0.019	0.42–3.49	1.25 (-0.19–2.68)	0.088
Metastatic disease	38.09 (14.13–62.04)	0.002	17.18–58.99	<0.001	17.99–57.27	32.84 (9.23–56.45)	0.007

OLS: ordinary least squares, as reported in Table 10 (R^2 = 0.191, adjusted R^2 = 0.158). Diagnostics: Shapiro-Wilk test of residuals $p < 0.001$; Breusch-Pagan test for heteroscedasticity $p = 0.570$; Durbin-Watson statistic 2.26; maximum variance inflation factor 1.02; maximum Cook's distance 0.133 (no observation above 1; 4 observations above the 4/n screening threshold); 2 observations with |standardized residual| > 3. HC3: heteroscedasticity-consistent standard errors. Bootstrap: percentile intervals from 2,000 resamples of cases. Median (quantile, $\tau = 0.5$) regression with asymptotic confidence intervals. Three patients with a baseline MEDD of 0 mg/day were excluded because the percentage change is undefined. CI: confidence interval; MEDD: morphine equivalent daily dose.

Table S7. Primary malignancies within the cancer-type categories used in the analysis.

Primary malignancy	n (%)
Gastrointestinal	61 (57.5)
Pancreas	37 (34.9)
Colorectal	14 (13.2)
Gastric	7 (6.6)
Hepatobiliary and other GI	3 (2.8)
Genitourinary	17 (16.0)
Bladder	5 (4.7)
Cervix	4 (3.8)
Prostate	3 (2.8)
Uterus/endometrium/vulva	3 (2.8)
Ovary	2 (1.9)
Lung	14 (13.2)
Lung and pleura	14 (13.2)
Other	14 (13.2)
Head and neck	4 (3.8)
Malignant melanoma	2 (1.9)
Hematologic	2 (1.9)
Intracranial	2 (1.9)
Breast	1 (0.9)
Neuroendocrine	1 (0.9)
Soft tissue	2 (1.9)

Percentages refer to the whole cohort (n = 106). Colorectal includes colon and rectal carcinoma; hepatobiliary and other gastrointestinal includes cholangiocarcinoma, gastrointestinal stromal tumor and peritoneal carcinomatosis; lung and pleura includes one pleural mesothelioma; head and neck includes oral cavity, gingival and parotid carcinoma; hematologic includes multiple myeloma and lymphoma.

Table S8. Morphine-equivalent conversion factors used to calculate morphine equivalent daily dose (MEDD).

Opioid	Route	Dose unit used	Conversion factor for MEDD
Morphine	Oral	mg/day	1
Oxycodone	Oral	mg/day	1.5
Tramadol	Oral	mg/day	0.2
Fentanyl	Transdermal	µg/h	2.4

Conversion factors follow the 2022 CDC Clinical Practice Guideline [20], as implemented in the Northwest Pain Guidance Opioid Conversion Calculator [19]. *For example, a 25 µg/h transdermal fentanyl patch corresponds to 60 mg/day MEDD (25 × 2.4). Methadone was not used in this cohort. MEDD: morphine equivalent daily dose.

Figure legends

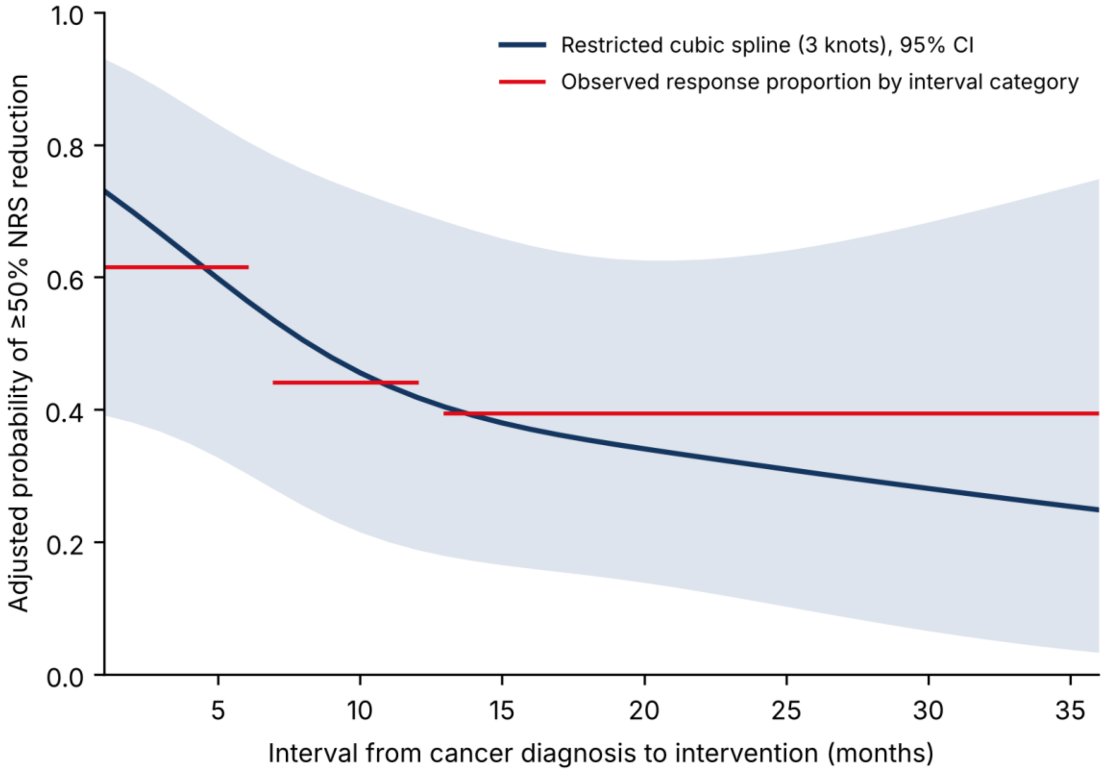

Figure S1. Adjusted probability of ≥50% NRS reduction at 1 month according to the interval from cancer diagnosis to intervention, modelled with a restricted cubic spline with three knots (navy line, shaded 95% confidence band; other covariates of the full model held at their observed values and averaged). Red horizontal segments show the observed response proportion within the exploratory interval categories (≤6, 7–12 and >12 months). p for non-linearity = 0.367; p for overall association = 0.013. The curve is truncated at 36 months because only three patients had longer intervals.

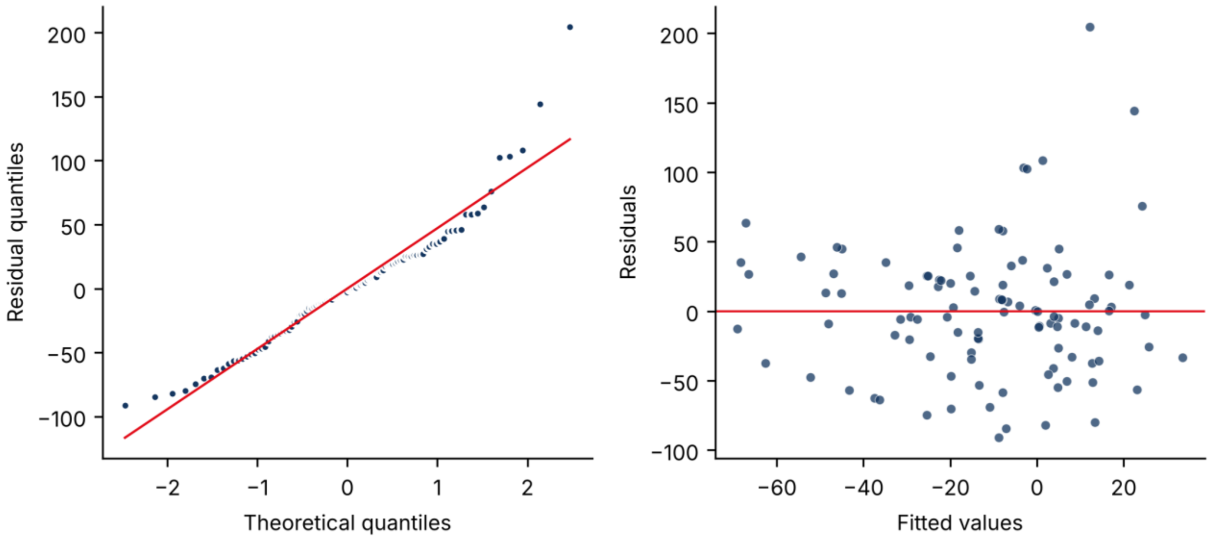

Figure S2. Residual diagnostics of the multivariable linear regression for percentage change in MEDD (Table 10). Left: normal quantile-quantile plot of residuals. Right: residuals against fitted values.
