# Supplementary material for: Association Between the Cancer Diagnosis-to-Intervention Interval and Short-Term Analgesic Response Following Interventional Pain Treatment: A Retrospective Cohort Study
Source: J Clin Med. 2026 Oct 16;15(20):8019. doi: 10.3390/jcm15208019 (PMC13706139; doi:10.3390/jcm15208019)
Supplement: Supplementary file 1 [file jcm-15-08019-s001.zip › jcm-4555018-supplementary-STROBE-checklist-v4-cohort.pdf]

STROBE Statement—Checklist of items that should be included in reports of ***cohort studies***

	Item No	Page
**Title and abstract**	1	Abstract file
		(*b*) abstract file
**Introduction**		
Background/rationale	2	1
Objectives	3	2
**Methods**		
Study design	4	2
Setting	5	3
Participants	6	(*a*) 3
		(*b*)
Variables	7	3
Data sources/ measurement	8*	3
Bias	9	4
Study size	10	3
Quantitative variables	11	3
Statistical methods	12	(*a*) 4
		(*b*) 4
		(*c*) 4
		(*d*) 4
		(*e*) 4
**Results**		
Participants	13*	(a) 5
		(b) 5
Descriptive data	14*	(a) 5
		(b) 5
		(c) 5
Outcome data	15*	5
Main results	16	(*a*) 5-7
		(*b*) 5-7
		(*c*) 5-7
Other analyses	17	5-7
**Discussion**		
Key results	18	8
Limitations	19	8-11
Interpretation	20	8-11
Generalisability	21	8-11
**Other information**		
Funding	22	On title page

*Give information separately for exposed and unexposed groups.

Note: An Explanation and Elaboration article discusses each checklist item and gives methodological background and published examples of transparent reporting. The STROBE checklist is best used in conjunction with this article (freely available on the Web sites of PLoS Medicine at http://www.plosmedicine.org/, Annals of Internal Medicine at

http://www.annals.org/, and Epidemiology at http://www.epidem.com/). Information on the STROBE Initiative is available at http://www.strobe-statement.org.
